# Intracultural variation of knowledge about wild plant uses in the Biosphere Reserve Grosses Walsertal (Austria)

**DOI:** 10.1186/1746-4269-8-23

**Published:** 2012-07-06

**Authors:** Christoph Schunko, Susanne Grasser, Christian R Vogl

**Affiliations:** 1 Working Group: Knowledge Systems and Innovations, Division of Organic Farming, Department for Sustainable Agricultural Systems, University of Natural Resources and Life Sciences (BOKU), Gregor-Mendel Straße 33, Vienna, 1180, Austria

## Abstract

**Background:**

Leading scholars in ethnobiology and ethnomedicine continuously stress the need for moving beyond the bare description of local knowledge and to additionally analyse and theorise about the characteristics and dynamics of human interactions with plants and related local knowledge. Analyses of the variation of local knowledge are thereby perceived as minimal standard. In this study we investigate the distribution and variation of wild plant knowledge in five domains: food, drinks, human medicine, veterinary medicine and customs. We assess relations between the wild plant knowledge of informants and their socio-demographic as well as geographic background.

**Method:**

Research was conducted in the Biosphere Reserve Grosses Walsertal, Austria. Structured questionnaires were used to inquire wild plant knowledge from 433 informants with varying socio-demographic and geographic background. Children assisted in the data collection. Data was analysed using descriptive statistics and generalized linear models.

**Results and discussion:**

A majority of respondents is familiar with wild plant uses, however to varying degrees. Knowledge variations depend on the socio-demographic and geographic background of the informants as well as on the domains of knowledge under investigation: women, older informants and homegardeners report more human medicinal applications and applications in drinks than men, younger informants and non-homegardeners; farmers know a greater variety of veterinary medicinal applications than non-farmers; the place of residence relates significantly to food and veterinary uses. Customs are difficult to investigate in standardized matrices. The household-related distribution of work and the general socio-cultural context are especially helpful in order to explain intracultural variation of knowledge in the Grosses Walsertal.

**Conclusions:**

Research on the intracultural variation of local knowledge exposes cultural characteristics and highlights the cultural embeddedness of local knowledge. The impact of socio-cultural developments on local knowledge may be anticipated from understanding the intracultural variation of knowledge.

## Background

Leading scholars in ethnobiology and ethnomedicine continuously stress the need for moving beyond the bare description of local knowledge (LK) and to additionally analyse and theorise about the characteristics and dynamics of human interactions with plants and related LK (e.g. [1-4]). Analyses of the variation of LK are thereby perceived as minimal standard [2,3,5]. Insights in the intracultural variation of knowledge help to identify the characteristics of more and less knowledgeable individuals [3], lead to hypotheses about the social organisation in a culture [6], give indications of persistence or loss of LK [7] and thereby help to identify the conditions for the thriving and vanishing of LK. Moreover, intracultural variation of knowledge is suggested to bear an important potential for informing adaptation processes in times of uncertainty [8].

So far, the intracultural variation of LK has been assessed in selected cultures and several regions of the world. . The type of knowledge and skills under investigation include plant species identification [7,9], plant classification [10], knowledge of pests [11], and the use of plant species in diverse use categories, such as food, medicine, building material, firewood or domestic goods (e.g. [12-14]). The variables tested relating to these domains and skills include age [7,10], gender [5,13], family background [11], modernization [12,15,16], culture [14], geography [14], market access [17], education [17] and plant accessibility [18].

Although there is some diversity among the domains investigated, many studies concentrate on the intracultural variation of local medicinal knowledge (LMK) (e.g. [19-22]). These studies find that age has a positive relation with LMK, and it is only found to diminish towards the end of one’s lifetime [22]. Gender differences in LMK are inconsistent between cultures and are suggested to depend, at least partly, on the cultural division of labour [22,23]. Higher education is found to reduce LMK [24], while occupation is found to have varying effects, depending on the kind of occupation [24]. Some studies also find that isolation is associated with high levels of LMK [25,26], whereas modernization is associated with low levels of LMK [21,27]. Other authors suggest that modernization does not influence LMK in this linear and straightforward manner (increasing modernization, decreasing LMK), but that the “nature of change may be subtle, complex, and specific to particular treatments” [24].

A few studies also compare the intracultural variation of knowledge concerning several different domains of knowledge [12-14,16]. These studies find that the relation between peoples and domains of knowledge are complex and multilateral. E.g. among the Rarámuri people in Mexico, women know more plant species for medicinal uses, men are more familiar with plant species used for making domestic goods and for construction and both sexes hold similar knowledge about edible plants and firewood [13]. Among the Roviana people of the Solomon Islands, modernization leads to increasing knowledge of the cash value of plant species, while it does not influence the knowledge in other domains [12]. In south-western Spain a decline of traditional agricultural knowledge in general but a persistence of traditional livestock farming knowledge can be identified [16]. And among the Shuhi people in southwest China, the accessibility to plant species influences not so much the selection of ritual and medicinal plant species, yet more the selection of plant species in other domains [18].

These results demonstrate the dynamic nature and intracultural variation of LK and show that the intracultural variation of LK is patterned following socio-demographic characteristics of informants, geographic characteristics as well as domains of knowledge. Reasons for the intracultural variation of LK are suggested to include the social organisation [12], distribution of work [13], and other cultural [14,16,20] as well as ecological factors [14,20].

Although several studies on the variation of LK were conducted in several parts of the world, very limited information is available for Europe (notable exceptions: [16,20]).

In this study we aim to (1) investigate the distribution and variation of wild plant knowledge relating to five domains of knowledge, (2) assess the relation of several socio-demographic and geographic variables to individuals’ wild plant knowledge, (3) identify reasons for the intracultural variation of knowledge, and (4) add European findings to the discussion on the intracultural variation of LK. The knowledge under investigation in this paper is commonly studied in ethnobiological research: wild plant knowledge. More precisely, our study concentrates on the respondents’ ability to recognise plant species under a shared name and to cite respective uses for every plant, independent from the respondents’ actual use, i.e. his or her behaviour.

While several of the variables and use categories chosen in this study were selected in former research (variables: *gender*, *age*, *geography*; use categories: *food*, *drink*, *human medicine*), we also investigated the variation of knowledge relating to variables and use categories unconsidered before (variables: *farming*, *homegarden maintenance*; use categories: *veterinary medicine*, *customs*).

## Methods

### Field site

**Figure 1:**
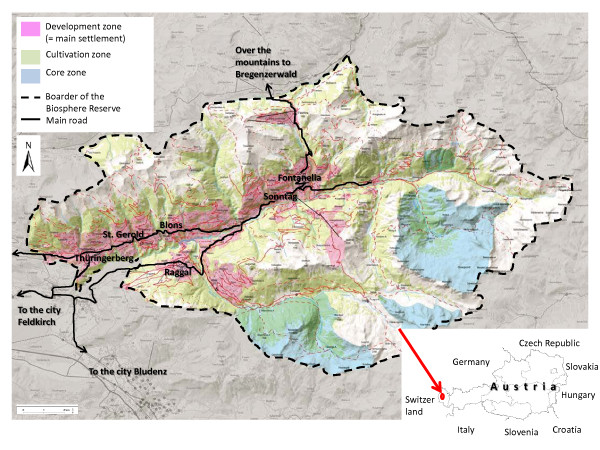
**Map of the Biosphere Reserve Grosses Walsertal (Source: [**[28]**], modified).**

The *Grosses Walsertal* (GWT) is a sparsely populated mountain valley characterized by alpine farming and situated in *Vorarlberg*, the very western province of Austria. Approximately 3,400 people live in an area of 192 km^2^ in the six municipalities of Thüringerberg, St. Gerold, Blons, Sonntag, Fontanella and Raggal [28]. Thüringerberg, St. Gerold, Blons and Raggal are located comparably closer to the capital of the district, Feldkirch, whereas the inhabitants of Sonntag and Fontanella are situated more remote, at the end of the valley. Thüringerberg, St. Gerold, Blons, Sonntag and Fontanella are located on the sunny side of the valley, whereas Raggal is located on the shady side (Figure 1).

The steep valley has only little even ground. It is characterized by flourishing green flysch mountains in the northern part and steep lime mountains in the southern part. The landscape is shaped by meadows and pastures due to long lasting livestock husbandry [28].

For centuries the people of this remote region were cut off almost entirely, which supported the creation and conservation of a distinct culture including a specific dialect [29].

There are 180 active farmsteads in the valley of which 40% are run organically [28]. About 37% of the inhabitants of the GWT work in the GWT – 16% in small trade enterprises, 11% in agriculture, 8% in tourism, and 3% in public service – whereas 61% commutes outwards the valley for work [30]. The importance of agriculture in the valley has decreased over the last decades and steadily less acreage is worked on by a decreasing number of farmers. However, farming has become intensified since the remaining farms work on more land and keep more livestock nowadays [30].

Since the year 2000, the GWT has been acknowledged as UNESCO Biosphere Reserve [28].

### Research process, sampling and questionnaire

Research for this study was conducted in two stages. During the first field research period, between July and September 2008, 36 interviews (34 women, 2 men) were conducted with recommended specialists using *freelists* and subsequent semi-structured interviews [31]. The sampling strategy applied was snowball sampling [31] and the freelist question was “Welche Pflanzen fallen dir ein, die hier im Tal wild wachsen und gesammelt werden?” (literal translation: “Which plants can you think of that grow wild in the valley and are gathered?”). The data collected during this field period allowed us to identify the most frequently known and used plant species in the GWT as well as to understand the domain of wild gathered plant species from an emic perspective. The concept of *wild plant species* was defined by the respondents and therefore all plant species listed as wild species by the respondents were accepted as such by the authors. The findings from the freelist interviews are reported in detail elsewhere [32].

The study at hand is based on data collected in spring 2009 during the second field research period, where we investigated the general distribution and intracultural variation of wild plant knowledge in the GWT. We therefore approached the inhabitants via the pupils of the seven primary schools in the valley.

The second author of the paper first contacted the heads of the primary schools to discuss the idea and later involved teachers and local actors in order to design the methods and organize the data collection. The project was announced in the local newsletter of the Biosphere Reserve. The parents of all partaking pupils were informed through a letter explaining the character of the project, including its partners, aims and anonymity of data, and in two schools the parents were also informed during school meetings. Every person displayed in the figures gave informed consent for publication.

The second author then organized wild plant workshops in the schools to prepare the pupils, aged six to ten, for the topic of wild plant gathering. At the end of these workshops she presented the questionnaire and subsequently asked the pupils to fill in the questionnaire with several family members separately as homework. Hence, the pupils represented the interviewers in this study (like done by e.g. [33] before; please also see our forthcoming article [34] where we discuss the issue of children as enumerators in detail). Every pupil received four copies of the questionnaire. The teachers were asked to collect the questionnaires, once filled in. This sampling strategy allowed us to gather information from a large number of people living in the GWT. However, it also created some bias since people without a connection to children aged six to ten were not reached.

The questionnaire used was close-ended and represented a matrix (rows: plant species; columns: use categories). The respondents were asked to tick, based on their knowledge, the appropriate use categories for each of the plant species listed. The plant species selected were the 20 most frequently mentioned wild gathered plant species elicited in the freelist interviews of the first field research period. The use categories chosen were food, drink, human folk medicine, veterinary folk medicine and customs, which were also the most frequently mentioned use categories in the freelist interviews [32]. In the questionnaire the respondents were additionally asked to state their gender, age, occupation, place of residence and if they work in a homegarden.

The questionnaire was designed in a child-oriented and simple way and e.g. included drawings. It was checked for its quality, practicability and comprehensibility in a pre-test with two children interviewing a parent or grandparent.

In total, 506 questionnaires (about 15% of the total population) were returned by 189 pupils. The 433 questionnaires which were filled in completely were used for further analyses. The sample consisted of 130 male and 303 female individuals ranging from 7 to 84 years of age (median: 41 years). Twenty-five per cent of the sample are homekeepers, 16% employees, 15% farmers, 13% pupils, 12% retirees, 10% labourers and 9% had other occupations. Twenty-five per cent of the sample live in Sonntag, 20% in Raggal, 18% in Thüringerberg, 14% in Fontanella, 11% in Blons and 11% in St. Gerold. Sixty-three per cent of the respondents work in a homegarden, whereas 37% do not (Table 1).

**Table 1:** Descriptive statistics of independent variables selected for Generalized Linear Models (n = 433)

**Variable**	**Value = 1**	**Mean**		
Sex Female	Female	.70		
Homegardening	Yes	.37		
Occupation Employee	Yes	.16		
Occupation Farmer	Yes	.15		
Entry to valley	Yes	.40		
Sunny side of valley	Yes	.80		
**Variable**	**Minimum**	**Maximum**	**Mean**	**Stand. Dev.**
Age	7	84	43.09	18.12

Data were entered for storage into an MS Access database [35] with support from local women.

### Remarks and limitations concerning methodology

LK is more than the number of mentioned items or ticked boxes and includes tacit knowledge, behavioural skills, practice of knowledge through social organization, specific world views and expression through language [36]. The research approach in this study focuses on one section of LK only, namely the ability of respondents to recognize plant species under a shared name and to cite respective uses for every plant name. The implementation of LK in behaviour is e.g. not presented.

We took several precautions to ensure good data quality and responsiveness of informants. These included developing the questionnaire together with teachers and local actors, a child-oriented design, pre-testing in a real setting, preparation workshops for children, pre-information by means of a local newsletter, an information letter for parents, and an information meeting with some parents. However, as is the case with many other methods where the investigator does not directly witness the collection of data, the children and their informants filled in the questionnaires without the attendance of a researcher.

Our research methodology does not allow determining whether answers were ticked intentionally or mistakenly. We therefore presume in the analysis that all given answers are correct. This becomes especially relevant in the case of very rare use reports since we cannot determine if these derive from specialist knowledge or errors.

Our study is biased towards women. During the freelist interviews in the first field research period mainly women were suggested for interviews through snowball sampling (34 women, 2 men). The 20 top ranked plant species, on which the data gathering in the second field research period is based, may therefore be biased towards plants and use categories especially known and used by women. Also in the second field research period the sample is biased towards women (303 women, 130 men), although the children were not influenced on which family member to interview. This indicates that women are more often perceived as knowledgeable about wild plants. The bias towards women may especially be reflected in the descriptive statistics whereas biases are neutralized in the models applied. However, women might still have had advantages due to the women-based selection of the 20 top ranked plant species.

### Data analysis

We first conducted descriptive analyses of the questionnaires and calculated how many per cent of all informants cited the plant uses proposed.

In a second step, we analysed the variation of LK through the creation of generalized linear models (GLM) using the linear type. These models basically calculate multiple regressions between several independent variables that are binomial, multinomial and/or categorical and a linear dependent variable [37]. The independent variables selected were socio-demographic and geographic variables and the dependent variables were the numbers of plant species cited per person as used in specific use categories (e.g. number of plant species used as food). Distinct models were created for every use category. Hence, e.g. the influence of the independent variables on the number of plant species used as food was tested in a separate model as well as the use in drinks, etc. Spurious relationships are neutralized in GLM. We only included main effects in the models, and chose Type III analyses and the Wald chi²-statistics [37].

The independent socio-demographic and geographic variables explored are gender (male/female), age (in years), homegarden cultivation (yes/no), occupation farmer (yes/no), occupation employee (yes/no) and two variables concerning the place of residence in the valley. These are location at the entry to the valley (entry: St. Gerold, Thüringerberg, Blons, Raggal; end: Fontanella, Sonntag) and location at the sunny side of the valley (sunny side: St. Gerold, Thüringerberg, Blons, Sonntag, Fontanella; shady side: Raggal) (Table 1). The use categories selected as dependent variables are food, drink, human medicine, veterinary medicine and customs. The usual 0.05 significance level is used to assess if an independent variable relates significantly to a dependant variable [31]. The regression coefficient B gives information about the strength of the relation and can be directly interpreted in linear models (e.g. for gender: if B = 2, women, on average, listed two plant species more than men in the respective use category; or for age: if B = 0.1, respondents, on average, listed 0.1 plant species more with every additional year of age). Subsequently we used descriptive statistics to examine our results more closely using inferential statistics, especially to clarify which plant species are primarily responsible for significant relationships in the data.

Descriptive analyses were carried out in PASW 18 [38] and MS Excel [39] and inferential statistics were performed in PASW 18 [38].

Project results were returned to the pupils through feedback workshops in each school class. Respondents of the survey were informed about the outcomes of the research through the local newsletter of the Biosphere Reserve [40].

## Results

### Distribution of knowledge

In total, 11,084 uses for the 20 plant species listed were indicated by the 433 respondents. Most uses were indicated for drinks (4,475 use reports), followed by human medicine (3,343 use reports), food (2,340 use reports), veterinary medicine (641 use reports) and customs (285 use reports). The average mean per questionnaire sums up to 26 use reports (standard deviation: 12). This includes on average ten use reports of drinks, eight use reports in human medicine, five use reports of foods, one use report in veterinary medicine and one use report in customs for every informant.

**Figure 2:**
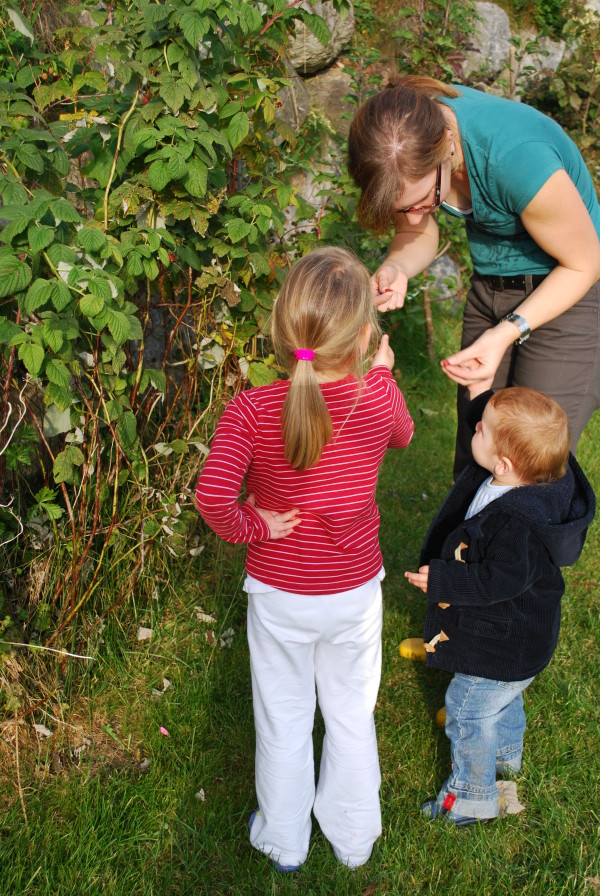
**Woman picking raspberries (*Rubus idaeus*) with her children (Photo: Susanne Grasser).**

The most frequently reported uses of wild gathered plant species, each indicated by more than 90% of the respondents, are *Rubus idaeus* and *Vaccinium myrtillus* for food and *Matricaria chamomilla* and *Mentha sp.* for drinking (Table 2, Figure 2). More than two-thirds of the respondents (67%–89%) reported the use of *Sambucus nigra* and *Taraxacum officinale agg.* for food, the use of *Alchemilla alpina*, *Alchemilla vulgaris*, *Salvia officinalis* and *Urtica dioica* for drinking, and the use of *Arnica montana* and *Calendula officinalis* in human folk medicine. And still mentioned by more than half of the respondents (51%–66%) were the use of *Achillea millefolium agg.*, *Primula sp., Rhododendron sp*., *Rubus idaeus* and *Sambucus nigra* for drinking and the use of *Hypericum perforatum* and *Salvia officinalis* in human folk medicine.

**Table 2:** Distribution of wild plant knowledge in the GWT concerning five use categories (n = 433)

**Plant species**	**Local Name**	**Use category (in per cent of all respondents)**				
		**Food**	**Drink**	Hmed*	Vmed**	**Customs**
*Abies alba/Picea abies*	Tanne	46	15	27	4	17
*Achillea millefolium agg.*	Schafgarbe	2	**61**	33	6	1
*Alchemilla alpina*	Frauenmantel	1	**81**	46	6	3
*Alchemilla vulgaris agg.*	Silbermantel	1	**76**	47	6	2
*Arnica montana*	Arnika	2	23	**74**	14	1
*Calendula officinalis*	Ringelblume	9	26	**78**	17	2
*Hypericum perforatum*	Johanniskraut	3	31	**65**	13	3
*Matricaria chamomilla*	Kamille	6	**91**	48	15	1
*Mentha sp.*	Pfefferminze	14	**91**	28	3	0
*Plantago lanceolata*	Spitzwegerich	10	46	46	6	1
*Primula sp.*	Schlüsselblume	13	**55**	21	4	6
*Rhododendron sp.*	Alpenrose	3	**52**	13	2	20
*Rubus idaeus*	Himbeere	**92**	**55**	24	3	1
*Salvia officinalis*	Salbei	20	**76**	**52**	5	2
*Sambucus nigra*	Schwarzer Holunder	**68**	**59**	33	3	1
*Taraxacum officinale agg.*	Löwenzahn	**70**	23	24	10	1
*Thymus serphyllum agg.*	Wilder Thymian	32	26	33	4	2
*Trifolium pratense*	Rotklee	18	25	11	11	1
*Urtica dioica*	Brennnessel	40	**84**	43	9	1
*Vaccinium myrtillus*	Heidelbeere	**91**	40	28	6	0

*Hmed Human medicine, **Vmed Veterinary medicine.

The use of the 20 selected wild gathered plant species in veterinary medicine and in customs was reported less frequently. The most frequently reported plant uses in these categories are *Calendula officinalis* (17% of the respondents), *Matricaria chamomilla* (15%), *Arnica montana* (14%) and *Hypericum perforatum* (13%) in veterinary medicine and of *Rhododendron sp.* (20%) and *Abies alba/Picea abies* (17%) in customs.

The plant species having the most use reports are *Urtica dioica* (765 use reports), *Rubus idaeus* (751 use reports), *Vaccinium myrtillus* (718 use reports), *Sambucus nigra* (708 use reports) and *Matricaria chamomilla* (698 use reports). The plant species reported least frequently used are *Trifolium pratense* (288 use reports), *Rhododendron sp*. (387 use reports), *Thymus serphyllum agg*. (421 use reports), *Primula sp*. (428 use reports) and *Achillea millefolium agg*. (442 use reports).

### Intracultural variation of knowledge

We identified ten significant relations between the seven independent and five dependent variables in the five models created (Table 3).

**Table 3:** Generalized Linear Models (GLM) showing effects of selected independent variables on number of plant species listed in distinct use categories (n = 433)

**Independent Variable**	**Food**		**Drink**		Hmed**		Vmed***		**Customs**	
	Regr B*	***p***	***Regr B***	***p***	***Regr B***	***p***	***Regr B***	***p***	***Regr B***	***p***
Sex Female	*.492*	*.054*	**2.164**	**.000**	**2.341**	**.000**	-.021	.946	-.192	.144
Age	*-.012*	*.066*	**.035**	**.001**	**.029**	**.034**	.000	.955	*-.006*	*.071*
Homegardening	.363	.139	**1.191**	**.004**	**1.883**	**.000**	*.555*	*.058*	*.245*	*.052*
Occupation Employee	.036	.911	-.186	.731	.068	.920	-.549	.153	-.120	.468
Occupation Farmer	-.430	.203	.809	.154	-.313	.658	**1.436**	**.000**	**-.378**	**.030**
Entry to valley	-.254	.338	-.594	.182	-.490	.376	-.187	.554	.019	.891
Sunny side of valley	**-.797**	**.012**	-.129	.809	.019	.977	**−1.063**	**.005**	-.207	.206

*Regression Coefficient B.

**Human medicine.

***Veterinary medicine.

Food uses were reported significantly more often by people living on the shady side of the valley than by people living on the sunny side (p = 0.012), although the relation is weak (B = −0.797). The relation of women and younger respondents ticking more food uses than men and older respondents was marginally not significant (p = 0.054 and p = 0.066 respectively). Wild plant uses in drinks and human medicine were reported significantly more often by women than by men (p < 0.001 for drinks and p < 0.001 for human medicine), by older respondents than by younger respondents (p = 0.001 for drinks and p = 0.034 for human medicine) and by homegardeners than by non-homegardeners (p = 0.004 for drinks and p < 0.001 for human medicine), whereby the relations are medium to strong (variable gender: B = 2.164 for drinks and B = 2.341 for human medicine; variable age: B = 0.035 for drinks and B = 0.029 for human medicine; variable homegardening: B = 1.191 for drinks and B = 1.883 for human medicine). Veterinary wild plant uses were reported significantly more often by farmers than by non-farmers (p < =0.001) and by people living on the shady side of the valley than by people living on the sunny side of the valley (p = 0.005), both having medium relations (B = 1.436 for occupation farmers; B = −1.063 for sunny side of the valley). Additionally, homegardening has a marginally not significant positive relation with veterinary wild plant uses (p = 0.058). Furthermore, wild plant uses in customs were reported more frequently by non-farmers than by farmers (p = 0.030), although the relation is very weak (B = −0.378), and marginally not significant more often by younger people than older people (p = 0.071) and by homegardeners than by non-homegardeners (p = 0.052).

Hence, each of the variables gender, age, homegarden maintenance, occupation farmer and living on the sunny side of the valley relate significantly to two different use categories while the variables occupation employee and living at the entry of the valley do not relate significantly to any use category.

The descriptive statistics showing which plant species are primarily responsible for significant relation in the data are not presented here due to their complexity (see Additional file 1). However, we use excerpts from these statistics in the discussion section to interpret some of the above results from inferential statistics.

## Discussion

### Distribution of knowledge

Several recent studies highlighted that knowledge about the use of wild plant species persists in many places in rural Europe (e.g. [41-43]). Whereas most studies interviewed specialists and older individuals (e.g. [5,41,44]), our results suggest that a large majority of the variety of people living in the GWT disposes of wild plant knowledge (Table 2).

Most plant uses in the GWT were reported for the use in drinks, human medicine and food and all frequently mentioned plant uses (mentioned by more than 50% of respondents) occur in these three use categories. Comparably few plant uses in veterinary medicine and in customs were stated.

The dominance of these three use categories is suggested to be a remnant from a strong history of subsistence in the valley and of the historic importance of wild foods, drinks and medicine [45]. The comparably high popularity of applications in drinks (mostly herbal teas with medicinal effects [32]) and human medicine may be linked with the formerly high degree of isolation of the people in the valley and the associated late arrival of modern healthcare. Compared to that, veterinary medicinal knowledge is less widespread since veterinary applications are only relevant for people who keep animals, mainly farmers (15% of informants). The low percentage of use reports of customs seems to be astonishing. For example, only 17% of the respondents reported *Abies alba/Picea abies* as used in customs, although probably almost everyone in the valley has a Christmas tree made from *Abies alba/Picea abies* at home at Christmas time, as it is a widespread tradition in Christian countries. We believe that this result stems from two different reasons: 1) interviews were conducted in summertime and few people thought about plant species used in customs during other periods of the year (such as e.g. Christmas); 2) most people perceived the category “customs” (*Brauchtum*) as big celebrations during the year (such as e.g. Easter, *Alpabtrieb* (bringing the livestock down from the alpine pastures),…) and did not associate the smaller everyday customs with the category although the other plant species selected for investigation might be used. Hence our results suggest that customs are a complex domain, which is hard to investigate in a standardized form.

The frequently listed wild plant uses in the GWT are also often mentioned in the relevant literature (e.g. [46]). However, evidence about the extent of the distribution of wild plant knowledge was rare before. Our findings show that four wild food uses, six uses in drinks and two uses in human medicine are known by more than two third of the respondents and therefore by an ample majority of the diverse people living in the GWT (Table 2). Further information about these plant uses are presented elsewhere [32].

### Intracultural variation of knowledge

For Europe only a few studies on the intracultural variation of plant knowledge are available. We therefore draw on worldwide literature to discuss our results.

#### Wild food uses are more frequently known by people living on the shady side of the valley

Descriptive statistics indicate that especially *Abies alba/Picea abies*, *Thymus serphyllum agg*., *Urtica dioica* and *Primula sp.* are reported as used for food more often on the shady side than on the sunny side of the valley (each plant species listed >10% more often by people living on the shady side) (see Additional file 1). These plant species grow in the wild and are available on both sides of the valley. We therefore do not believe that geographical reasons are responsible for this knowledge variation but suggest that socio-cultural reasons cause this relationship. Most likely a group of people with close social ties, e.g. a neighbourhood or a circle of families or friends, might be interested in using wild food plants and therefore might know the use of these plant species especially often. The weak correlation (people living on the shady side listed on average less than one food plant more than people living on the sunny side) indicates that the differences between people living on the sunny and shady side of the valley are moderate though.

#### Uses in drinks and human medicine are reported more often by women, older persons and people working in homegardens

The society of the GWT has a strong agricultural heritage and life in the GWT still follows traditional patterns of rural life in Europe. Men thereby used to be in charge of the physically demanding agricultural work while they frequently accept employment and work away from home today. Women used to fulfill agricultural and non-agricultural tasks in the household and around the home and many women in the valley today work as homekeepers (in our sample: 108 out of 303 women or 36% of all women (including pupils and retirees) interviewed). Tasks of women in former times and still today are preparing food, maintaining health and treating illnesses of family members. We suggest that this distribution of work influences knowledge variation in the GWT and that women reported more plant uses for human medicine and drinks (and marginally not significant more food uses) because they are in charge of food and medicine related tasks in the households. These relations are the strongest ones identified in the data (every woman surpasses every man by more than two use reports in drinks and human medicine on average), which further highlights the influence of the distribution of work on knowledge patterns. Distribution of work also relates to knowledge variation among the Rarámuri people in Mexico where women know more medicinal plants and also have a major responsibility in harvesting them. On the other hand men are more familiar with plants used for construction and for making domestic goods and they also have a major role in harvesting and working with these plants [13]. In the GWT men report less wild plant uses in two, almost three, out of the five selected domains. However, we need to consider the gender bias towards women in our data collection and a more gender balanced selection of plant species and domains might have valorised wild plant knowledge of men. But the frequent recommendation of (field research period one) and orientation towards women (field research period two) when it comes to wild plant knowledge also shows that women are perceived more knowledgeable about wild plants.

Older people reported more uses of wild plant species in human medicine and related drinks than younger individuals (with every additional 34 (human medicine) and 29 (drinks) years of age respectively, respondents report one plant species more). This may indicate that (1) medicinal knowledge, which was important in former times of subsistence where doctors were not immediately available, degrades or that (2) medicinal knowledge of wild plant species gets acquired only later in life, when physical inconveniences often increase. Looking at the declining importance of subsistence in the GWT [45] we are inclined to accept the first argument. However, we agree that definite conclusions on the dynamics of human medicinal knowledge within lifetimes and across generations can only be drawn on the basis of longitudinal data, received through observation over time [2,6].

**Figure 3:**
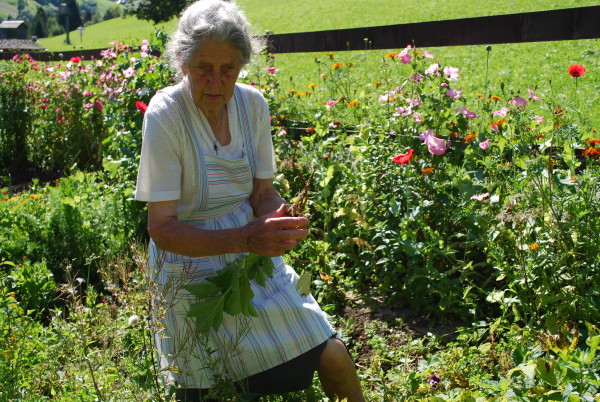
Elderly woman working in her homegarden (Photo: Susanne Grasser).

Homegardens are frequently maintained for increasing the subsistence of households (e.g. [47]). This concerns food subsistence, but our results show that homegardeners also report more wild plant uses in human medicine and drinks (Figure 3). Homegardeners in the GWT seem to adopt concepts of subsistence which do not only include food production but also useful knowledge for maintaining health.

In contrast to that, reports about wild food uses were similar between homegardeners and non-homegardeners, a fact that indicates a popularity of wild foods (in contrast to wild medicinal plants) also for non–homegardeners.

Another reason for the obvious knowledge surplus of homegardeners might be that several of the investigated wild plant species are also commonly grown in homegardens (e.g. *Calendula officinalis*, *Mentha sp.*, *Salvia officinalis*, *Matricaria chamomilla*[32]), and homegardeners may therefore know these plant species and their virtues better than non–homegardeners. Descriptive statistics confirm this interpretation since three out of the four plant species reported much more often by homegardeners than non–homegardeners for the use in drinks are also common garden plants (*Calendula officinalis, Primula sp., Rubus idaeus*) (each plant species listed >10% more often by homegardeners) (see Additional file 1). And in human medicine, homegardeners reported the use of fourteen plant species, also including garden plants, much more often than non–homegardeners (each plant species listed >10% more often by homegardeners) (see Additional file 1). Hence, our results suggest that homegardeners report more wild plant uses about human medicine and drinks because (1) homegardeners include maintaining health in their concept of subsistence and/or because (2) homegardening enhances knowledge about wild plant uses when plant species are both cultivated and harvested from the wild.

#### Veterinary medicinal applications are more often known by farmers and people living on the shady side of the valley

**Figure 4:**
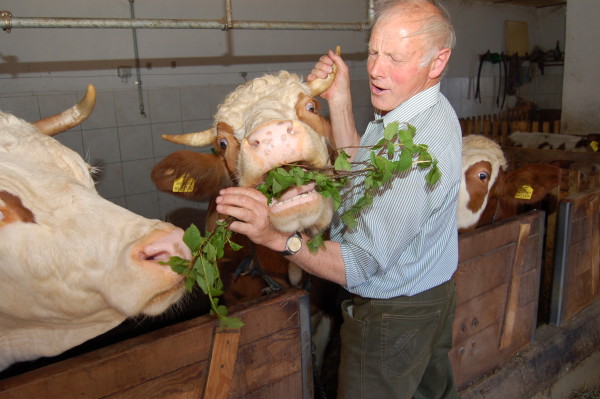
Farmer delivering a tree branch as remedy to one of his cows (Photo: Susanne Grasser).

We expected the first part of this result since most farmers in the GWT keep animals (mainly milk cows and cattle) and use ethnoveterinary applications to treat illnesses of their livestock (Figure 4) [48].

The interpretation of the second part of this result is more challenging. Part of the explanation might be that more organic farmers live on the shady side of the valley than on the sunny side and that those organic farmers get advice and are also obliged by the European Organic Farming Law to use natural medicines and treatments [49]. However, further research is needed to illuminate this result.

#### Plant uses in customs are more frequently mentioned by non-farmers than by farmers

**Figure 5:**
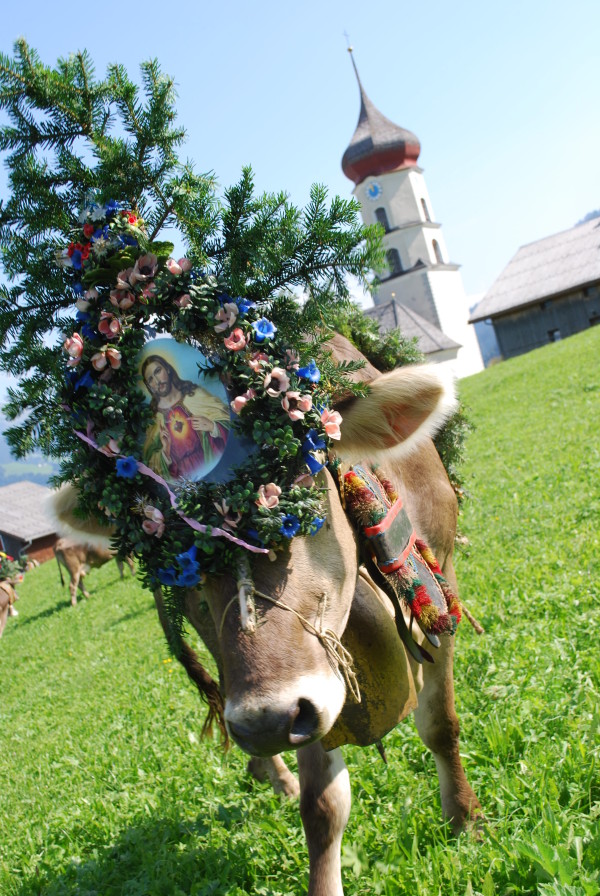
Cow decorated with Picea abies (among others) for the custom of Alpabtrieb (bringing the livestock down fromthe alpine pastures) (Photo: Susanne Grasser).

Although only a very weak relation was identified (non-farmers reported on average about 0.4 plant species more than farmers), this result is astonishing since former research in western Austria found that farmers use a variety of wild and home-grown plant species in customs, mainly as ornamental plants (Figure 5) [47,50]. We therefore see three possible explanations for this result: (1) a different understanding of the use category customs by farmers and non-farmers, (2) farmers are less aware of the customs they live or (3) farmers actually exert fewer customs than non-farmers.

The plant species less known as used by farmers are especially *Rhododendron sp.* (reported by 21% of non-farmers and 14% of farmers) and *Abies alba/Picea abies* (reported by 18% of non-farmers and 12% of farmers) (see Additional file 1). Both plant species are well known in the valley and have widespread uses in customs [32], which easily surpass the investigated percentages of listed uses. We are therefore more inclined to accept explanations (1) and (2) than explanation (3). This result again points to the fact that customs are difficult to investigate in a standardized matrix.

#### Occupation employee and living at the entry/end of the valley does not relate to knowledge about wild plant uses

Although farmers relate significantly to use categories, employees do not, and occupation therefore does not *per se* relate to wild plant knowledge. However, special occupations can relate to more knowledge in specific domains, like in our case occupation farmer and the domain of veterinary medicinal knowledge. Hence, occupation can or cannot relate to more knowledge of informants in ethnobotanical domains, depending on the kind of occupation and the domain in question. Similar results were found in rural Dominica, where commercial occupation tended to increase medicinal knowledge while higher education tended to reduce this kind of knowledge [24].

We tested living at the entry of the valley for its relation to knowledge of informants since the differences in remoteness of the villages are considerable in the GWT and remoteness was found to relate to knowledge levels in earlier publications (e.g. [26]). However, location at the entry to the valley was found to have no significant relation to knowledge in our case. Thus, although some villages in the GWT are geographically more remote than others, this does not influence knowledge of informants, probably since the connectedness to modern life is quite the same all over the valley nowadays.

## Conclusions

People living in the GWT generally hold wild plant knowledge, but the kind and amount of such knowledge varies with their socio-demographic background, geographic location and the domain of knowledge. Hence, no general statements about variation of wild plant knowledge should be made in future research and at least a differentiation between domains of knowledge should be considered.

Some socio-demographic characteristics of people relate to more knowledge variation than others. We identified gender, age, homegardening, occupation farmer and location at the sunny side of the valley as influential variables, while occupation employee and living at the entry to the valley were not influential. While gender, age, occupation and location were considered as having relations to the variation of plant knowledge in past research, homegardening was unconsidered so far. Our results suggest that homegardening is substantially related to the acquisition and storage of plant knowledge and we believe that future research should make efforts to further investigate the potential role of homegardening for the propagation and conservation of plant knowledge.

The distribution of work in households and the general socio-cultural context is especially helpful for explaining intracultural variation of knowledge. This points to the cultural embeddedness of LK and highlights that socio-cultural changes will very likely result in changes in the intracultural variation and general availability of LK. Research on the intracultural variation of knowledge demonstrates this embeddedness and helps to understand dynamics of knowledge as well as to anticipate the impact of socio-cultural developments on LK. Local activists and policy makers may use insights from research on the intracultural variation of knowledge to conserve and propagate selected domains of LK.

## Competing interests

The authors declare that they have no competing interests.

## Authors’ contributions

SG designed the methods approach and carried out field work. CS conducted quantitative data analysis, composed the literature review and drafted the manuscript. SG supplemented the draft. CRV substantially assisted in all stages of this study. All authors read and approved the final manuscript.

## Supplementary Material

Additional file 1 Plant species and their frequency of use relating to use categories and variables selected. Click here for file
